# Nosocomial bloodstream infections in a teaching hospital in Vietnam: a five-year analysis

**DOI:** 10.1017/ash.2025.161

**Published:** 2025-09-03

**Authors:** Pham Thi Lan, Pham Thi Truong Ngan, Nguyen Vu Hoang Yen, Trinh Thi Thoa, Truong Thi Le Huyen, Huynh Hoang Hai, Le Thanh Truyen, Nguyen Thi Minh Khai, Le Thị Yen Nhi, Le Mong Hao, Huynh Minh Tuan

**Affiliations:** 1 University Medical Center, HCM city; 2 University of Medicine and Pharmacy at HCM city

**Keywords:** Bloodstream infection, Intensive care unit, Healthcare-associated infection

## Abstract

**Introduction:** Nosocomial Bloodstream infection (BSI), including central line-associated blood stream infection (CLABSI) is important causes of morbidity and mortality. There are few studies describing the epidemiology of BSI in Viet Nam. **Methods:** A cross-sectional descriptive study was conducted in 3 intensive care units (ICUs) of the University Medical Center (UMC), Ho Chi Minh City from 2017 to 2022. The UMC service microbiology database was accessed to identify positive blood culture specimens during the period 2017–2022. Demographic and clinical details, antimicrobial management and patient outcome information were extracted from medical and laboratory records. **Results:** Of the 695 unique bacterial and fungal BSI episodes identified during the study period, 232 (33.4%) were community-acquired (CA), and 463 (66.6%) hospital-acquired (HA). The rate of BSI was 11.4% (463 cases/4.069 patients), in which CLABSI accounted for 59.8%. The incidence of CLABSI was 13.2% (307 cases/2.320 catheter patients) and the incidence rate was 5.8 cases per 1.000 catheter-days. On multivariable analysis, severe underweight, patient origin, central line placed in the femoral vein, duration catheter-days were significantly associated with CLABSI. We observed that prolonged duration catheter were the main risk CLABSI with 2.7- fold for 14-28 cathter-days (OR=2.7, 95% CI 2.4-3.1), 7.3-fold for more than 28 catheter-days (OR=7.3, 95% CI 5.7-9.4). The most common organisms were Gram-negative bacteria (76.2%), with *K. pneumoniae* (31.4%) and *A. baumannii* (12%) most prevalent. Gram-negative bacteria and Candida were more likely to cause infections in patients in critical care units. In addition, patients with BSI had significantly greater ICU costs than patients with Non-BSI (422 million VND (IQR 239–680) vs 184 million VND (IQR 18–92), p <0.05) **Conclusions:** Our data suggest that catheter duration is an important risk factor for CLABSI in the ICU. A significant daily increase in the risk of CLABSI after 28 days may warrant CVC replacement if intravascular access is necessary beyond that period.

